# Expression of *Francisella* pathogenicity island protein intracellular growth locus E (IglE) in mammalian cells is involved in intracellular trafficking, possibly through microtubule organizing center

**DOI:** 10.1002/mbo3.684

**Published:** 2018-07-05

**Authors:** Takashi Shimizu, Shiho Otonari, Jin Suzuki, Akihiko Uda, Kenta Watanabe, Masahisa Watarai

**Affiliations:** ^1^ The United Graduate School of Veterinary Science Yamaguchi University Yamaguchi Japan; ^2^ Joint Faculty of Veterinary Medicine Laboratory of Veterinary Public Health Yamaguchi University Yamaguchi Japan; ^3^ Department of Veterinary Science National Institute of Infectious Diseases Shinjuku Japan

**Keywords:** *Francisella*, MTOCs, type VI secretion system

## Abstract

*Francisella tularensis* is the causative agent of the infectious disease tularemia and is designated a category A bioterrorism agent. The type VI secretion system encoded by the *Francisella* pathogenicity island (FPI) is necessary for intracellular growth; however, the functions of FPI proteins are largely unknown. In this study, we found that the FPI protein intracellular growth locus E (IglE) showed a unique localization pattern compared to other FPI proteins. Deleting *iglE* from *Francisella tularensis* subsp. *novicida* (*F. novicida*) decreased intracellular growth. Immunoprecipitation and pull‐down assays revealed that IglE was associated with β‐tubulin. Additionally, GFP‐fused IglE colocalized with microtubule organizing centers (MTOCs) in 293T cells. The *iglE* deletion mutant was transferred with dynein toward MTOCs and packed into lysosome‐localizing areas. Conversely, the wild‐type *F. novicida* exhibited intracellular growth distant from MTOCs. In addition, IglE expressed in 293T cells colocalized with dynein. These results suggest that IglE helps to prevent dynein‐ and MTOC‐mediated intracellular trafficking in host cells to inhibit the transport of *F. novicida* toward lysosomes.

## 1 INTRODUCTION

Effector proteins are virulence factors secreted by various pathogenic bacteria. Effector proteins hijack intracellular signaling and trafficking and the cytoskeleton, changing the intracellular environment to promote bacterial replication and survival (Mattoo, Lee, & Dixon, 2007). These effectors are secreted by various secretion systems, which are classified into nine types (types I–IX) (Desvaux, Hébraud, Talon, & Henderson, 2009; Nakayama, 2015). Because they play a critical role in virulence, effector proteins, and secretion systems are attractive targets for the treatment of infectious diseases (Charro & Mota, 2015). Of the nine secretion systems, only the type VI secretion system (T6SS) is present in members of the genus *Francisella* (Bröms, Sjöstedt, & Lavander, 2010). Although several *Francisella* effector proteins are reportedly important for its intracellular growth and virulence (Bröms, Meyer, Sun, Lavander, & Sjöstedt, 2012; Eshraghi et al., 2016), the molecular mechanisms underlying the functions of these proteins are poorly understood.

*Francisella tularensis* subsp. *tularensis* (*F. tularensis*) is a gram‐negative, facultative intracellular bacterium that causes the zoonotic disease tularemia (Decors et al., 2011; Ellis, Oyston, Green, & Titball, 2002), and it is classified as a category A bioterrorism agent (Maurin, 2015). The vector‐borne transmission of *F. tularensis* to mammalian hosts precedes the development of tularemia (Petersen, Mead, & Schriefer, 2009; Suzuki, Uda, Watanabe, Shimizu, & Watarai, 2016; Suzuki, Hashino, et al., 2016). *F. tularensis* subsp. *novicida* (*F. novicida*) is closely related to *F. tularensis* and is also a facultative intracellular pathogen that replicates within macrophages (Anthony, Burke, & Nano, 1991). Although *F. novicida* exhibits low virulence in humans, it is thought to share considerable homology with *F. tularensis* and thus serves as a practicable surrogate for studies (Kingry & Petersen, 2014).

*Francisella* are ingested through the pseudopod loops of macrophages and taken up into spacious vacuoles possessing endosomal markers (Clemens, Lee, & Horwitz, 2004, 2005). Subsequently, the bacteria escape from the phagosomes and replicate in the cytosol (Golovliov, Baranov, Krocova, Kovarova, & Sjöstedt, 2003). In the late stage of infection, the bacteria reenter the autophagosomes (Checroun, Wehrly, Fischer, Hayes, & Celli, 2006). They acquire amino acids from degraded proteins and replicate there (Checroun et al., 2006). Replication‐deficient or damaged cytosolic bacteria are caught by lysosomal‐associated membrane protein 1 (LAMP‐1)‐positive autophagosomes, termed *Francisella‐*containing vacuoles and are degraded by the ubiquitin‐SQSTM1‐LC3 pathway (Chong et al., 2012).

The *Francisella* pathogenicity island (FPI) is a genetic element that is critical for the intracellular growth of *Francisella* (Barker et al., 2009; Nano et al., 2004). *F. tularensis* possesses two copies of the FPI, whereas *F. novicida* contains a single copy. The FPI is a cluster of approximately 30 kb in length, coding 16–19 open reading frames. Although the sequence homology is limited, several FPI genes are thought to encode a T6SS (Bröms et al., 2010; Clemens, Ge, Lee, Horwitz, & Zhou, 2015), one of the secretion systems recently discovered in gram‐negative bacteria (Cianfanelli, Monlezun, & Coulthurst, 2016; Ho, Dong, & Mekalanos, 2014; Mougous et al., 2006; Pukatzki et al., 2006). The T6SS injects protein effectors into host cells or neighboring bacteria (Hachani, Wood, & Filloux, 2016) through a contractile needle similar to a T4‐like bacteriophage tail (Aksyuk et al., 2009). The T6SS is critical for quorum sensing, stress responses, biofilm formation, symbiosis, virulence, intramacrophage growth, and antibacterial activities of pathogenic bacteria such as *Burkholderia mallei*,* Pseudomonas aeruginosa*,* Vibrio cholera*, and *Campylobacter jejuni* (Chen, Zou, She, & Wu, 2015; Lertpiriyapong et al., 2012; Pukatzki et al., 2006; Schwarz et al., 2010). However, the detailed functions of FPI proteins in host cells have yet to be described.

In this study, we carried out an expression analysis of FPI proteins and found that the *F. novicida* intracellular growth locus E (IglE) shows unique localization and is associated with microtubule‐organizing centers (MTOCs) to modulate membrane trafficking for the intracellular growth of the bacterium.

## 2 MATERIALS AND METHODS

### 2.1 Bacterial strains and culture conditions

*F. novicida* U112 was obtained from the Pathogenic Microorganism Genetic Resource Stock Center (Gifu University). *F. novicida* was cultured aerobically at 37°C in a chemically defined medium (CDM) (Nagle, Anderson, & Gary, 1960) or in a brain–heart infusion broth (Becton, Dickinson and Company, Franklin Lakes, NJ) supplemented with cysteine (BHIc) (Mc Gann et al., 2010) containing 1.5% agar (Wako Laboratory Chemicals, Osaka, Japan).

### 2.2 Cell culture

THP‐1 cells (human monocytic cell line) were grown in RPMI 1640 medium (Sigma‐Aldrich, St. Louis, MO) supplemented with 10% heat‐inactivated fetal bovine serum (FBS) at 37°C in an atmosphere containing 5% CO_2_; 293T cells (human kidney cell line) were grown in DMEM medium (Sigma‐Aldrich) supplemented with 10% heat‐inactivated FBS at 37°C in an atmosphere containing 5% CO_2_.

### 2.3 Plasmid construction, transformation, and transfection

Table S1 shows the primer sets and templates used to construct plasmids used in this study. PCR was carried out using KOD‐Plus‐Neo polymerase (Toyobo, Osaka, Japan), and ligation was performed with the Ligation High Ver. 2 kit (Toyobo) or the In‐Fusion HD Cloning Kit (Takara Bio, Otsu, Japan). Plasmids were transformed into *F. novicida* by cryotransformation (Pavlov, Mokrievich, & Volkovoy, 1996). Briefly, bacterial cells were suspended in transfer buffer (0.2 M MgSO4, 0.1 M Tris acetate [pH 7.5]) with 1 μg of plasmid DNA. The bacterial cells were frozen in liquid nitrogen, thawed at room temperature, and then cultured in CDM. Then, bacterial cells were collected and cultured on BHIc plates containing 50 μg/ml kanamycin or 2.5 μg/ml chloramphenicol. Plasmids were transferred into cell lines with FuGENE HD (Promega, Madison, WI) according to the instruction manual.

### 2.4 Construction of *F. tularensis iglE* and *dotU* mutants

The Δ*dotU* mutants of *F. novicida* were generated by group II intron insertion using the TargeTron Gene Knockout System (Sigma‐Aldrich) modified for *Francisella* species (Rodriguez, Yu, Davis, Arulanandam, & Klose, 2008), as described previously (Uda et al., 2014). Briefly, 2 μg of each pKEK‐DotU was transformed, and bacterial cells were precultured in CDM at 30°C for 6 hr. Then, the cells were collected and cultured on BHIc plates containing 50 μg/ml kanamycin at 30°C. Mutagenesis was confirmed using PCR to detect the 915‐bp insertion. To remove the plasmids, mutants cells were further cultured on BHIc plates without antibiotics at 37°C.

The Δ*iglE* mutant was constructed by homologous recombination. To make the suicide vector pFRSU, the promoter region of sacB and antibiotic‐resistance marker of pSR47s (Merriam, Mathur, Maxfield‐Boumil, & Isberg, 1997) were replaced with the *bfr* promoter of pNVU1 (Uda et al., 2014) and the kanamycin‐resistance gene *kanR* from pKEK1170 (Rodriguez et al., 2008), respectively. The upstream and downstream regions of *iglE* (1.5 kb each) were cloned into the *Sal*I site of pFRSU to make pFRSU‐IglE. One microgram of pFRSU‐IglE was transformed into *F. novicida*, and the cells were cultured on BHIc plates containing 50 μg/ml kanamycin. Isolated bacteria were cultured in CDM without antibiotics for 6 hr and then plated on BHIc plates containing 5% sucrose. The deletion of the *iglE* gene was confirmed by PCR.

### 2.5 GFP‐, mCherry‐, and IglE‐expressing *F. novicida* strains

The *gfp* gene with the Shine‐Dalgarno (SD) sequence was recloned from pGreenTIR (Miller & Lindow, 1997) into pNVU1 to make pNVU‐GFP. Then, the *gfp* gene with the *bfr* promoter of pNVU‐GFP was cloned into pOM5 (Pomerantsev, Obuchi, & Ohara, 2001) to make pOM5‐GFP. To make pOM5‐mCherry, the *gfp* gene of pOM5‐GFP was exchanged with *mCherry* gene from pmCherry‐C1 (Takara Bio). Chromosomal *iglE* from *F. novicida* genome was cloned into pNVU‐1 to make pNVU‐IglE. Then, the *iglE* gene with the *bfr* promoter from pNVU‐IglE was cloned into pOM5 to make pOM5‐IglE. To make the GFP and IglE double‐expressing plasmid pOM5‐GFP‐IglE, the *iglE* gene from pOM5‐IglE was inserted downstream of the *gfp* gene in pOM5‐GFP. pOM5‐GFP, pOM5‐mCherry, pOM5‐IglE, and pOM5‐GFP‐IglE were transformed into the wild‐type or Δ*iglE* mutant of *F. novicida* by cryo‐transformation.

### 2.6 IglE expression assay

The *iglE* gene from *F. tularensis* subsp. *tularensis* SCHU P9 (Uda et al., 2014) was cloned into pAcGFP‐C1 and pmCherry‐C1 to make pAcGFP‐C1‐IglE and pmCherry‐C1‐IglE. pAcGFP‐C1‐IglE or pmCherry‐C1‐IglE were transfected into 293T cells, using FuGENE HD. After 48 hr of incubation, the cells were treated and observed with confocal microscopy.

### 2.7 Immunoblotting

To generate an antiserum against IglE, rabbits were immunized with the C+TGKNEFPLDKDIKD peptide. The peptide and antiserum were prepared by Eurofine Genetics (Tokyo, Japan). *F. novicida* was cultured in CDM containing 5% KCl to an OD_595_ of 0.25. The culture supernatants were desalted to remove KCl with Amicon Ultra filters (Merck Millipore, Billerica, MA) and concentrated fivefold. Samples were mixed with SDS sample buffer (Thermo Fisher Scientific, Waltham, MA). Fifteen microliters of sample was loaded onto a NuPAGE Novex 4%–12% Bis‐Tris Gel (Thermo Fisher Scientific) and separated by SDS‐PAGE. Separated proteins were transferred onto a polyvinyl difluoride (PVDF) membrane (Merck Millipore). The membrane was treated with anti‐IglE antiserum (1:100) or anti‐PdpC antibody (Chong et al., 2008; Uda et al., 2014), a generous gift from Dr. J. Celli, followed by the treatment with HRP‐conjugated anti‐rabbit IgG (ab6717, 1:20,000; Abcam, Cambridge, UK). Proteins were detected with the ECL Prime Western Blotting System (GE Healthcare, Buckinghamshire, UK) and the LAS‐4000 mini Imaging System (Fujifilm Life Science, Tokyo, Japan).

### 2.8 Intracellular growth assay

THP‐1 cells (4 × 10^5^ cells/well) were preincubated in a 48‐well tissue culture plate with 100 nM of phorbol myristate acetate (PMA) for 48 hr. *F. novicida* strains were added at a multiplicity of infection of 1. These plates were centrifuged for 10 min at 300× *g* and incubated for 30 min at 37°C. Then, THP‐1 cells were washed twice with RPMI1640 medium, and extracellular bacteria were killed with a 60‐min gentamicin (50 μg/ml) treatment. To measure the intracellular growth of *F. novicida*, the THP‐1 cells were incubated in fresh medium at 37°C for the indicated time, washed three times with phosphate‐buffered saline (PBS), and then lysed with 0.1% Triton X‐100 in CDM. Colony‐forming units were determined by serial dilution on BHIc plates.

### 2.9 Fluorescence microscopy

293T cells (1 × 10^5^ cells/well) were transfected with 250 ng/well of pAcGFP‐C1‐IglE, pmCherry‐C1‐IglE, pAcGFP‐IglE‐N, or pAcGFP‐IglE‐C on 12‐mm glass coverslips (Matsunami Glass Industries, Ltd., Osaka, Japan). The cells were incubated for 48 hr and treated for microscopy. THP‐1 cells (4 × 10^5^ cells/well) were preincubated in a 48‐well tissue culture plate with 100 nM PMA for 48 hr on 12‐mm glass coverslips. *F. novicida* strains were infected similarly. After incubation for the indicated times, THP‐1 cells were treated for microscopy.

For MTOC staining, the cells were fixed with 4% paraformaldehyde at room temperature for 30 min and permeabilized with 0.1% TritonX‐100 in PBS. The cells were treated with anti‐pericentrin antibody (ab4448, 1:100; Abcam) and then stained with Alexa555‐conjugated anti‐rabbit IgG or FITC‐conjugated anti‐rabbit IgG (1:1,000). Fluorescent images were obtained with a FluoView. For LAMP‐1 staining, cells were fixed with the PLP Solution Set (Wako Laboratory Chemicals) containing 5% sucrose for 1 hr at 37°C and permeabilized with cold methanol for 10 s. The cells were treated with anti‐LAMP‐1 antibody (ab25245, 1:100; Abcam) and were stained with TRITC‐conjugated anti‐rat IgG (ab7094, 1:1,000; Abcam). To visualize endosomes, 100 μg/ml of Texas Red‐labeled dextran (molecular weight: 70,000; Thermo Fisher Scientific) was added to the cells 6 hr after transfection. For dynein staining, cells were fixed with 4% paraformaldehyde at room temperature for 30 min and permeabilized with 0.1% TritonX‐100 in PBS. Then, cells were treated with anti‐dynein antibody (ab23905, 1:20; Abcam) and stained with FITC‐conjugated anti‐mouse IgG (1:1,000). The FluoView FV100 confocal laser‐scanning microscope (Olympus, Tokyo, Japan) was used to obtain fluorescent images.

### 2.10 Immunoprecipitation assay

For the immunoprecipitation assays, 293T cells (1 × 10^6^ cells/well) in a six‐well plate were transfected with 2 μg/well of pAcGFP‐C1‐IglE, using FuGENE HD. After 48 hr of incubation, GFP‐fused proteins were collected, using the GFP‐Trap (ChromoTek GmbH, Planegg‐Martinsried, Germany) according to the instruction manual. Samples were suspended in SDS sample buffer and heated at 70°C for 15 min. Fifteen microliters of sample was loaded onto a NuPAGE Novex 4%–12% Bis‐Tris Gel, and the proteins that co‐precipitated with IglE‐GFP were separated by SDS‐PAGE followed by staining with Quick‐CBB PLUS (Wako Laboratory Chemicals). Peptide mass fingerprinting was performed according to the method of Yoshino et al. (Yoshino, Oshiro, Tokunaga, & Yonezawa, 2004). Briefly, the CBB‐stained bands obtained from SDS‐PAGE were excised and sliced into small strips. To remove the CBB, the strips were incubated in 50% methanol and 5% acetic acid for 1 hr and washed twice with water. The strips were dehydrated by incubation with 100% acetonitrile. To alkylate the proteins, the strips were incubated at 60°C for 1 hr with 10 mM dithiothreitol in 100 mM ammonium hydrogen carbonate followed by treatment at room temperature for 30 min with 55 mM iodoacetamide (Nacalai Tesque, Kyoto, Japan) in 100 mM ammonium hydrogen carbonate. In‐gel trypsin digestion was performed by incubating a gel with 10 μg/ml trypsin (Promega). The digested peptides were eluted using 5% formic acid (Wako Laboratory Chemicals). The peptides were desalted with ZipTip C18 Pipette Tips (Merck Millipore), spotted onto sample plates, and mounted with saturated α‐cyano‐4‐hydroxycinnamic acid (Nacalai Tesque) in 50% acetonitrile and 0.1% trifluoroacetic acid. An Autoflex mass spectrometer (Bruker Daltonics, Billerica, MA, USA) was used to measure the molecular weights of peptides. The reference database was searched by MASCOT software (Science Matrix, London, UK).

### 2.11 Pull‐down assay

The *iglE* gene from *F. tularensis* subsp. *tularensis* SCHU P9 was cloned into pCMV‐Myc‐N to make pCMV‐Myc‐N‐IglE. The β‐tubulin gene (*TUBB*) was cloned from THP‐1 cDNA into pCMV‐HA‐N to make pCMV‐HA‐N‐β‐tubulin. 293T cells (1 × 10^6^ cells/well) in a 6‐well plate were transfected with 2 μg/well of pCMV‐Myc‐N‐IglE or pCMV‐HA‐N‐β‐tubulin using FuGENE HD. After 48 hr of incubation, cells were lysed with 3 ml of lysis buffer (5 mM EDTA, 0.5% Triton X‐100, in PBS) containing protease inhibitor cocktail (Nacalai Tesque). Twenty microliters of protein G agarose beads (Cell Signaling Technology, Danvers, MA) was added to 1.2 ml of lysate and incubated for 1 hr at 4°C. Protein G agarose beads were removed, and 25 μl of protein G agarose beads and 2 μg of HA‐Tag polyclonal antibody (Takara Bio) were added. The lysate was incubated overnight at 4°C. Beads were collected by centrifugation, washed 5 times with PBS, and resuspended in 25 μl of SDS sample buffer (Thermo Fisher Scientific). Samples were heated at 70°C for 15 min and the beads were removed. Fifteen microliters of each supernatant was loaded on to a NuPAGE Novex 12% Bis‐Tris Gel and separated by SDS‐PAGE. The separated proteins were transferred to a PVDF membrane. The membrane was treated with 2 μg/ml of c‐Myc monoclonal antibody (Takara Bio) and HRP‐conjugated anti‐mouse IgG (1:10,000). Proteins were detected with the ECL Prime Western Blotting System (GE Healthcare) and the LAS‐4000 mini Imaging System.

### 2.12 Statistical analysis

One‐way analysis of variance was used to compare the results expressed as the means and standard deviations. Differences between the groups were determined by multiple comparisons using the Bonferroni/Dunnett method. The differences were considered significant at *p* values < 0.01.

## 3 RESULTS

### 3.1 IglE shows unique localization

**Figure 1:**
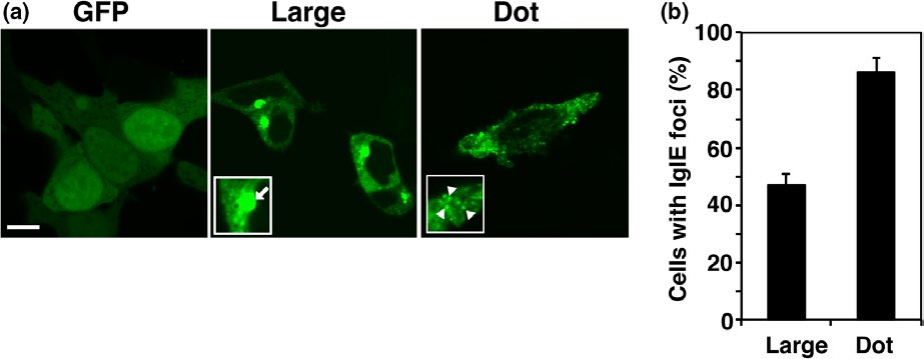
Intracellular growth locus E (IglE) shows unique localization. (a) 293T cells were transfected with pAcGFP‐C1‐IglE and incubated for 48 hr. The white arrow and arrowhead indicate large and dot foci, respectively. Scale bar: 20 μm. (b) The numbers of cells with large and dot IglE foci were calculated for 50 IglE‐expressing cells

Among the FPI proteins, 8 of them (PdpA, IglE, VgrG, IglF, IglI, IglJ, PdpE, and IglC) are considered to be secreted into the host cytosol (Bröms et al., 2012). To elucidate the function of FPI proteins secreted by *F. novicida*, we performed a comprehensive expression analysis of these eight FPI proteins. We assayed the localization of these proteins in host cells by expressing GFP fused to FPI proteins in 293T cells (Figure S1). Among the 8 FPI proteins, only IglE showed unique localization—large foci near the nucleus and dot foci (Figure 1a). The fractions of cells containing large or dot foci were 46.7 ± 4.2% and 86.0 ± 5.3%, respectively (Figure 1b).

### 3.2 Intracellular replication of *F. novicida* depends on IglE secretion

**Figure 2:**
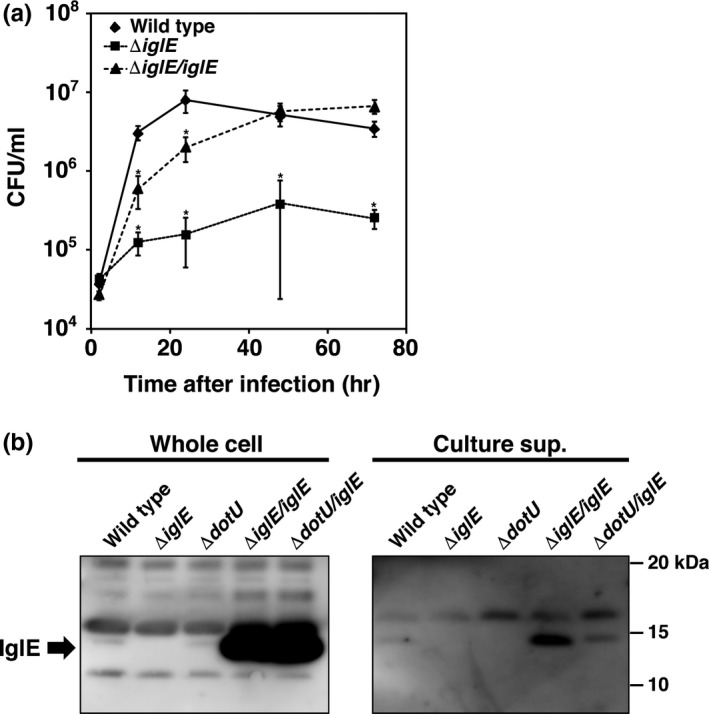
Intracellular replication of *Francisella novicida* is dependent on intracellular growth locus E (IglE) secretion. (a) THP‐1 cells were infected with *F. novicida* at multiplicity of infection = 1 and treated with 50 μg/ml of gentamicin. Cells were disrupted with 0.1% TritonX‐100 and plated on brain heart infusion broth supplemented with cysteine (BHIc) agar at indicated times postinfection. (b) *F. novicida* was cultured in BHIc medium‐containing 5% KCl. Bacterial cells were collected by centrifugation, and the supernatants were filtered and desalted with an ultrafiltration membrane. The presence of IglE in whole cells and in the culture supernatants was detected by immunoblotting

We focused on IglE as an effector protein and assessed its characteristics because of its unique localization. First, we constructed an *iglE* deletion (Δ*iglE*) mutant of *F. novicida* by homologous recombination. This mutation decreased the intracellular growth of *F. novicida* in THP‐1 cells, but complementation with wild‐type *iglE* restored the intracellular growth (Figure 2a). These results indicated that IglE was important for intracellular growth. To examine whether IglE protein was secreted into the culture medium, we cultured *F. novicida* in medium containing high concentrations of potassium chloride to mimic the host intracellular environment. Although the secretion of PdpC, an FPI protein, was not observed (Figure S2), we observed IglE secretion in the wild‐type and IglE overexpressing strains. However, the amount of IglE secreted from the wild‐type bacteria was limited. Importantly, the secretion was not observed in the *dotU* deletion mutant (Δ*dotU*, a gene encoding part of the T6SS apparatus), and the secretion decreased in the Δ*dotU* mutant that overexpressed IglE (Figures 2b and S2a).

### 3.3 IglE is associated with β‐tubulin and MTOCs

**Figure 3:**
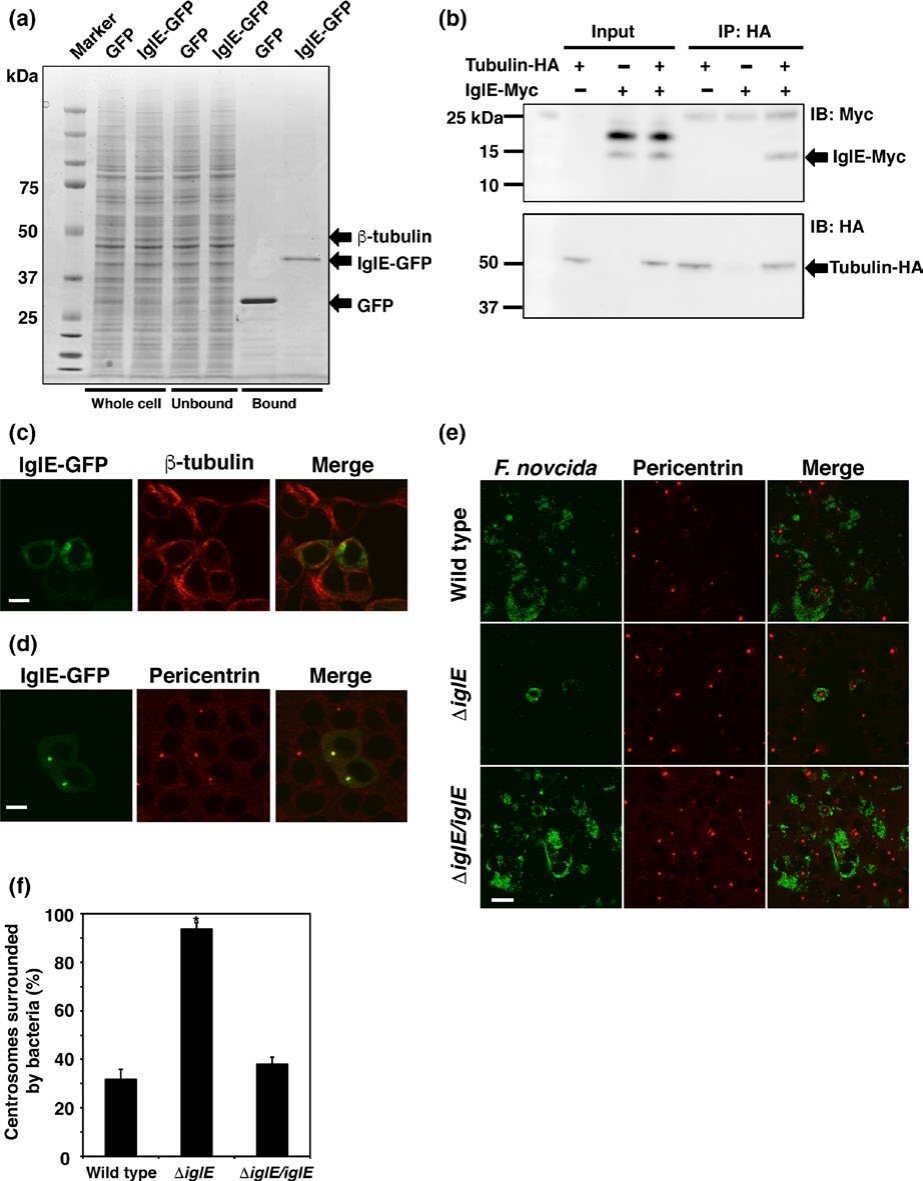
Intracellular growth locus E (IglE) is associated with β‐tubulin and microtubule organizing centers (MTOCs). (a) 293T cells were transfected with pAcGFP‐C1‐IglE and incubated for 48 hr. The cells were disrupted, and IglE‐GFP protein was precipitated with the GFP‐Trap. Co‐precipitated proteins were separated by SDS‐PAGE and extracted from the gel. The extracted protein was examined with matrix‐assisted laser‐desorption ionization/time‐of‐flight mass spectrometry. (b) Binding of IglE and β‐tubulin was confirmed with a pull‐down assay. 293T cells were transfected with pCMV‐HA‐N‐β‐tubulin and pCMV‐Myc‐N‐IglE. β‐tubulin was precipitated by anti‐HA antibody‐conjugated agarose beads and separated by SDS‐PAGE. Co‐precipitated IglE‐Myc protein was detected by immunoblotting for anti‐Myc antibody (IB: Myc). (c) 293T cells were transfected with pAcGFP‐C1‐IglE and incubated for 48 hr. β‐tubulin was stained using Alexa555‐conjugated anti‐β‐tubulin antibody. Scale bar: 20 μm. (d) 293T cells were transfected with pAcGFP‐C1‐IglE and incubated for 48 hr. MTOCs were stained using anti‐pericentrin antibody and Alexa555‐conjugated anti‐rabbit antibody. To observe the detailed localization of GFP‐fused IglE, the sensitivity of detection for GFP was decreased compared to the experiment in Figure 1a. Scale bar: 20 μm. (e) THP‐1 cells were infected with *Francisella novicida* harboring pOM5‐GFP at multiplicity of infection = 1 and treated with 50 μg/ml of gentamicin. At 24 hr postinfection, the cells were treated with anti‐pericentrin antibody and stained with Alexa555‐conjugated anti‐rabbit IgG. Scale bar: 40 μm. (f) The number of cells with MTOCs surrounded by *F. novicida* was calculated for *F. novicida*‐infected cells. *p < 0.01

To identify IglE‐binding proteins, GFP‐fused IglE was expressed in 293T cells, and GFP protein was precipitated with GFP‐binding protein‐conjugated agarose beads. The co‐precipitated proteins were separated by SDS‐PAGE. A protein of approximately 50 kDa was co‐precipitated with GFP‐fused IglE (Figure 3a). We identified this 50 kDa protein by matrix‐assisted laser‐desorption ionization/time‐of‐flight mass spectrometry as β‐tubulin. To confirm the interaction between IglE and β‐tubulin, a pull‐down assay was conducted. HA‐tagged β‐tubulin and Myc‐tagged IglE were expressed in 293T cells and precipitated with anti‐HA antibody using protein G agarose beads. Co‐precipitated Myc‐tagged IglE was detected by immunoblotting with an anti‐Myc antibody. Consequently, Myc‐tagged IglE was co‐precipitated with HA‐tagged β‐tubulin (Figure 3b). To determine whether IglE localized with β‐tubulin or microtubules in vivo, GFP‐fused IglE was expressed in 293T cells, and the cells were stained with an anti‐β‐tubulin antibody. Unexpectedly, we did not observe colocalization of β‐tubulin and IglE (Figure 3c).

Because β‐tubulin is a component of MTOCs (Conduit, Wainman, & Raff, 2015), we examined the localization of IglE and MTOCs. GFP‐fused IglE was expressed in 293T cells, and MTOCs were stained with an antibody against the MTOC marker pericentrin. The localization of MTOCs overlapped with the large foci of IglE (Figure 3d). To investigate the relationship between MTOCs and *F. novicida* infection, THP‐1 cells were infected with the wild‐type and Δ*iglE F. novicida*, and we assayed the localization of MTOCs (Figure 3e). When the host cells were infected with the wild‐type strain, bacteria were not observed around MTOCs. In contrast, Δ*iglE* mutants accumulated around MTOCs. In *F. novicida*‐infected cells, 93.8 ± 2.5% of MTOCs were surrounded by Δ*iglE* mutants, whereas only approximately 31.8 ± 4.1% and 38.1 ± 2.9% of MTOCs were localized with the wild‐type and complemented strains, respectively (Figure 3f). These results suggest that IglE interacts with MTOCs in host cells and inhibits the intracellular trafficking of *F. novicida* toward MTOCs.

### 3.4 IglE disturbs membrane trafficking through MTOCs

**Figure 4:**
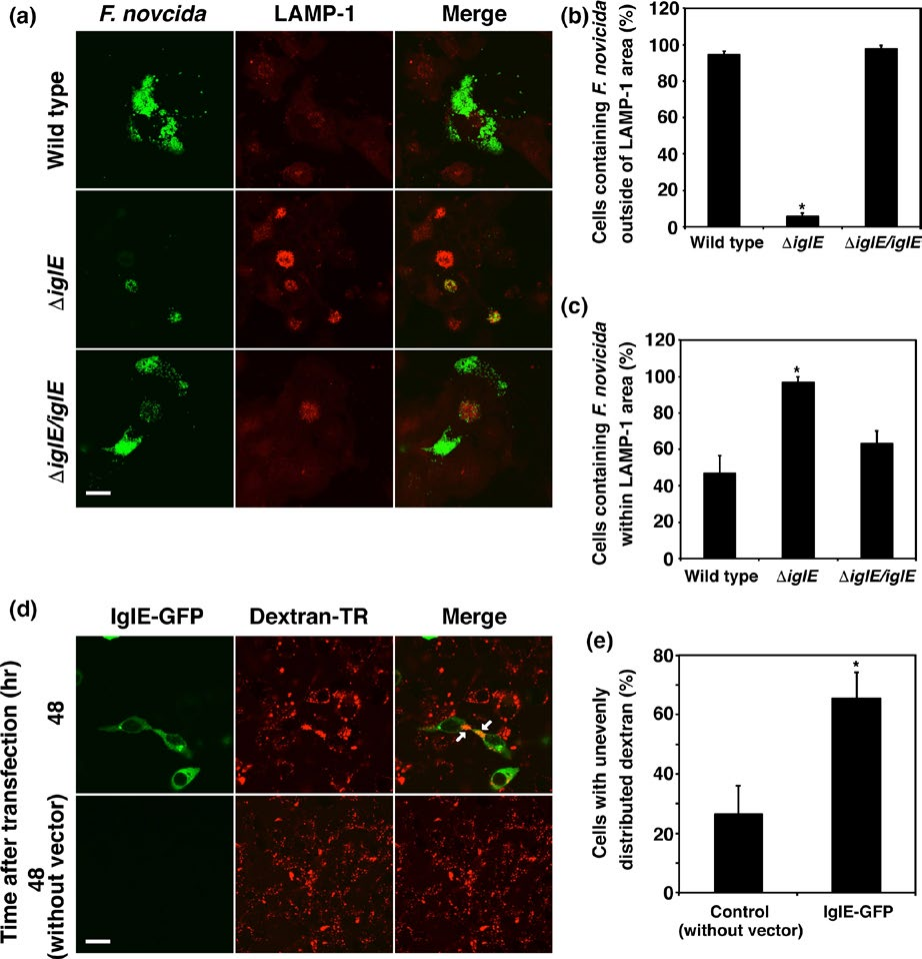
Intracellular growth locus E (IglE) disturbs membrane trafficking through microtubule organizing center. (a) THP‐1 cells were infected with *Francisella novicida* harboring pOM5‐GFP at multiplicity of infection = 1 and treated with 50 μg/ml of gentamicin. At 24 hr after infection, cells were treated with anti‐lysosomal‐associated membrane protein 1 (LAMP‐1) antibody and stained with TRITC‐conjugated anti‐rat IgG. Scale bar: 40 μm. (b) The number of cells containing *F. novicida* outside the LAMP‐1‐positive area was calculated for *F. novicida* infected cells. *p < 0.01. (c) The number of cells containing *F. novicida* colocalized to the LAMP‐1‐positive area was calculated for *F. novicida*‐infected cells. *p < 0.01. (d) 293T cells were transfected with pAcGFP‐C1‐IglE in the presence of dextran‐TR, and incubated for 48 hr. The white arrow indicates aggregated dextran. Scale bar: 20 μm. (e) The number of cells with aggregated dextran was calculated for cells with large IglE foci. *p < 0.01

In general, phagosomes are transported toward MTOCs on microtubules. Lysosomes are also present around MTOC and fuse with phagosomes (Blocker, Griffiths, Olivo, Hyman, & Severin, 1998). To examine the influence of IglE on this step, we infected THP‐1 cells with *F. novicida* and used an anti‐LAMP‐1 antibody to visualize the location of lysosomes. After infection, Δ*iglE* mutants localized within the LAMP‐1‐positive area, whereas the wild‐type and complemented strains grew in areas distant from the LAMP‐1‐positive area (Figure 4a). The fractions of THP‐1 cells containing *F. novicida* distant from a LAMP‐1‐positive area were 94.4 ± 1.9% for cells infected with the wild‐type bacteria and 97.7 ± 1.9% for cells infected with *iglE*‐complemented bacteria, whereas cells containing Δ*iglE* mutants distant from the LAMP‐1‐positive area numbered 5.6 ± 1.9% only (Figure 4b). Although most of the wild‐type and complemented bacteria did not localize to the LAMP‐1 positive area, some bacteria did accumulate in the LAMP‐1‐positive area (Figure 4a). Indeed, 46.7 ± 10.0% and 63.3 ± 6.7% of THP‐1 cells infected with the wild‐type or complemented bacteria contained bacteria within the LAMP‐1 positive area, respectively (Figure 4c).

To elucidate the effect of IglE on membrane trafficking, GFP‐fused IglE was expressed in 293T cells, and these cells were incubated with Texas Red‐labeled dextran. After 48 hr of incubation, the ingested dextran particles were distributed uniformly throughout the cytosol. However, in IglE‐expressing cells, dextran particles were not ingested (Figure 4d) or nonuniformly distributed and aggregated on one side (Figure 4d, white arrow). In cells with large foci near the nucleus, dextran particles were aggregated or not ingested in 65.6 ± 8.6% of cells (Figure 4e). These results suggest that IglE disturbs membrane trafficking in host cells by interacting with MTOCs, allowing *F. novicida* to escape from fusion with lysosomes.

### 3.5 IglE inhibits dynein‐based membrane trafficking

**Figure 5:**
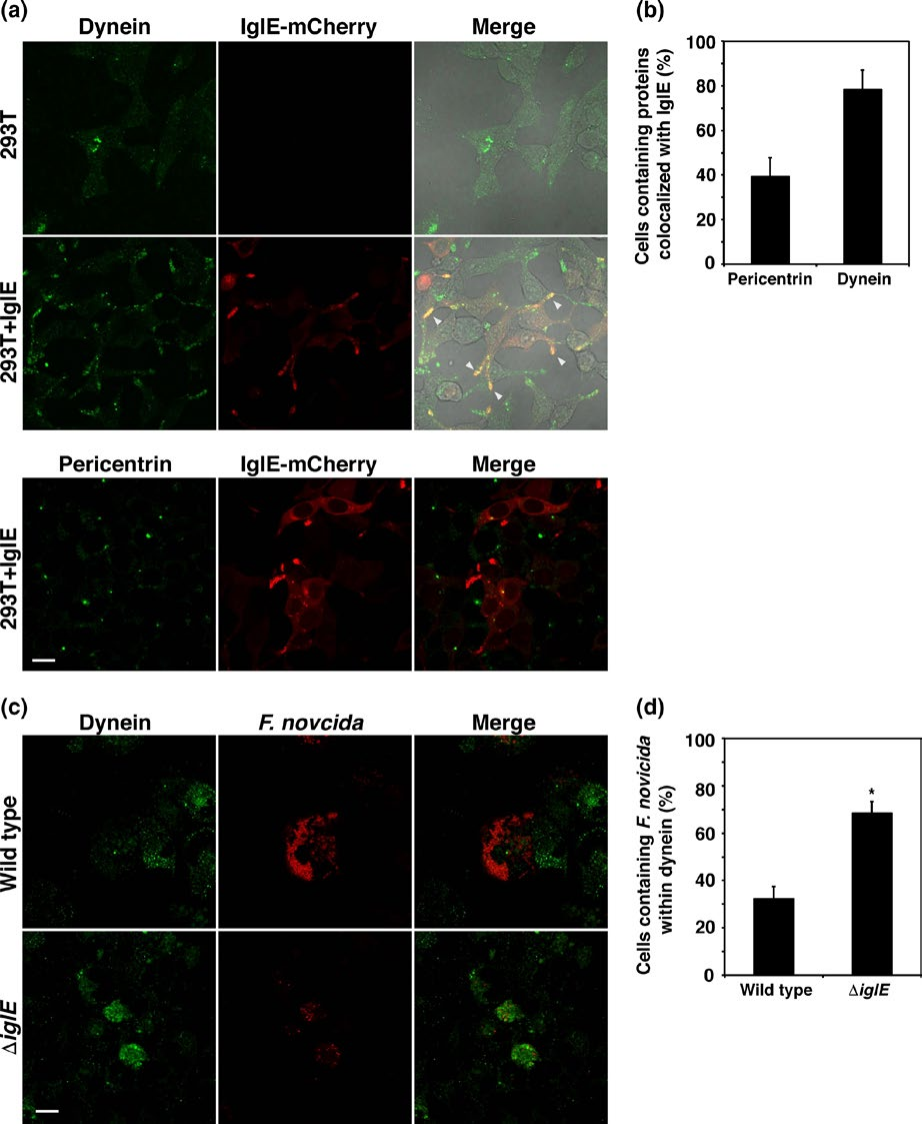
Intracellular growth locus E (IglE) colocalizes with dynein and disturbs dynein‐based membrane trafficking. (a) 293T cells were transfected with pmCherry‐C1‐IglE and incubated for 48 hr. Dynein was stained using anti‐dynein antibody and FITC‐conjugated anti‐mouse antibody. Fluorescent images were merged with differential interference contrast microscopy images. Microtubule organizing center (MTOC) was stained with anti‐pericentrin antibody and FITC‐conjugated anti‐rabbit IgG. Arrowheads indicate colocalization of IglE and MTOC. Scale bar: 40 μm. (b) The number of cells containing dynein or pericentrin colocalized with IglE was calculated for IglE‐expressing cells. (c) THP‐1 cells were infected with *Francisella novicida* harboring pOM5‐mCherry at multiplicity of infection = 1 and treated with 50 μg/ml of gentamicin. At 24 hr after infection, the cells were treated with anti‐dynein antibody and stained with FITC‐conjugated anti‐mouse IgG. Scale bar: 40 μm. (d) The number of cells containing *F. novicida* colocalized to the dynein‐positive area was calculated for *F. novicida*‐infected cells. *p < 0.01

Because the minus‐end of microtubules is located near the MTOC, IglE may inhibit membrane trafficking toward the minus‐end of microtubules. To confirm this hypothesis, we assayed the localization of the motor protein dynein, which moves toward the minus‐end of microtubules. In 293T cells‐expressing mCherry‐fused IglE, dynein was localized to the tips of cells and colocalized with the dot foci of IglE (Figure 5a). Among IglE‐expressing cells, 78.3 ± 8.8% of cells contained dynein colocalized with IglE (Figure 5b). In 293T cells‐containing mCherry‐fused IglE, IglE also colocalized with pericentrin (Figure 5a). In 39.4 ± 8.4% of IglE‐expressing cells, IglE colocalized with pericentrin (Figure 5b). In *F. novicida*‐infected THP‐1 cells, most wild‐type bacteria did not colocalize with dynein (Figure 5c), whereas in THP‐1 cells infected with the Δ*iglE* mutant, dynein accumulated and colocalized with the bacteria (Figure 5c). Among the cells infected with the wild‐type or Δ*iglE* mutant, 32.2 ± 5.4% and 68.3 ± 5.0%, respectively, contained bacteria within dynein‐positive areas (Figure 5d). These results indicate that IglE inhibits the dynein‐based trafficking of *F. novicida* toward MTOCs.

## 4 DISCUSSION

The molecular mechanisms underlying the actions of effector proteins from *Francisella* species are poorly understood. In this study, we revealed the function of the T6SS effector protein IglE, which associated with MTOCs and modulated the membrane trafficking of host cells. To identify how IglE affects the intracellular environment, we analyzed IglE‐binding proteins and found that β‐tubulin and pericentrin were associated with IglE. Pericentrin is a component of the γ‐tubulin ring complex (γ‐TuRC). The γ‐TuRC is the functional core of the MTOC and acts as a scaffold or a template for α/β‐tubulin dimers (Conduit et al., 2015). In THP‐1 cells, the *ΔiglE* mutant of *F. novicida* was transported to MTOCs, where lysosomes are located. Some bacterial effectors were reported to interact with microtubules or MTOCs and control the intracellular trafficking of bacteria. In *Salmonella enterica*, some T3SS effectors such as SseF or SseG interact with microtubules to form *Salmonella*‐induced filaments (Müller, Chikkaballi, & Hensel, 2012). In *Pseudomonas aeruginosa*, the T6SS effector VgrG2b associates with the γ‐TuRC, facilitating internalization of the bacterium (Sana et al., 2015). The *Chlamydia trachomatis* T3SS effector IPAM interacts with the MTOC protein CEP170 and controls microtubule assembly for inclusion morphogenesis (Dumoux, Menny, Delacour, & Hayward, 2015). Our findings suggest that IglE associates with MTOCs and controls membrane trafficking on microtubules. Indeed, membrane trafficking in IglE‐expressing cells was disturbed: dynein colocalized with IglE at the tips of cells, and ingested dextran particles accumulated on one side of the cells. *Francisella* species are ingested through phagocytosis and grow into the cytosol or in autophagosomes after they escape from phagosomes (Checroun et al., 2006; Chong et al., 2012; Clemens et al., 2004, 2005; Golovliov et al., 2003). For the maturation of phagosomes, endosomes and autophagosomes, motor‐based migration on microtubules toward the cell center, where lysosomes are located, is necessary (Blocker et al., 1998; Harrison, Bucci, Vieira, Schroer, & Grinstein, 2003; Kimura, Noda, & Yoshimori, 2008). In THP‐1 cells infected with the *F. novicida* Δ*iglE* mutant, bacteria accumulated with dynein around MTOCs where the lysosome marker LAMP‐1 was located, whereas the wild‐type bacteria were located far away from MTOCs. Together, these results imply that IglE associates with MTOCs to inhibit the trafficking of *F. novicida*‐containing phagosomes on microtubules and their subsequent fusion with lysosomes. This may allow *F. novicida* to escape from phagosomes and grow in the cytosol or in autophagosomes.

With a microscopic observation, IglE seemed to colocalize with MTOCs or dynein. Although IglE co‐precipitated with β‐tubulin, MTOC proteins such as pericentrin or dynein were not detected by the co‐precipitation assay with IglE. This may due to the abundance of β‐tubulin in cell cytosol. In addition, IglE expression in 293T cells failed to inhibit the depolymerization or repolymerization in the presence or absence of colchicine, an inhibitor of tubulin polymerization (data not shown). Therefore, the direct target of IglE is still unclear. However, IglE is expected to associate with β‐tubulin through MTOC or dynein because the colocalization of IglE and β‐tubulin was not observed with microscopy.

Several reports indicate that IglE is a bacterial lipoprotein with a signal peptide and is located at the bacterial membrane where it forms part of the T6SS apparatus (Bröms, Meyer, & Sjöstedt, 2017; Nguyen, Gilley, Zogaj, Rodriguez, & Klose, 2014; Robertson, Child, Ingle, Celli, & Norgard, 2013). In addition, we observed limited secretion of IglE into the culture medium. Thus, our results suggest that IglE may not be the so‐called effector protein. In fact, the secretion of other effectors, such as IglC, is inhibited in the Δ*iglE* mutant (Bröms et al., 2017). Therefore, in the case of the Δ*iglE* mutant, we could not rule out the possibility that its transportation to MTOCs and the inhibition of its intracellular growth were due to other effectors or a combination of IglE and other effectors. However, under our condition of IglE overexpression in host cells, IglE was associated with MTOCs and disturbed intracellular trafficking. These results at least suggest that IglE may have effector‐like functions when bacteria escape into the cytosol and IglE is exposed, or if bacteria are lysed and IglE is released into the cytosol. Because IglE had a signal peptide and was still secreted in the Δ*dotU* mutant that overexpressed IglE, IglE may be secreted by an unknown Sec protein‐related secretion system. Indeed, IglE is detected in the cytosol during *Francisella* infection (Bröms et al., 2012).

IglE could be a therapeutic target for treating *Francisella* infections or a biological tool for inhibiting intracellular trafficking because our results suggest that IglE affects MTOCs and modulates intracellular trafficking.

## CONFLICT OF INTEREST

All contributing authors declare no conflicts of interest.
