# Supplementary material for: Expression of Francisella pathogenicity island protein intracellular growth locus E (IglE) in mammalian cells is involved in intracellular trafficking, possibly through microtubule organizing center
Source: Microbiologyopen. 2018 Jul 5;8(4):e00684. doi: 10.1002/mbo3.684 (PMC6460260; doi:10.1002/mbo3.684)
Supplement: Supplementary file 1 [file MBO3-8-e00684-s001.docx]

TABLE S1

FIGURE S1

FIGURE S1 FPI proteins show unique localization. 293T cells were transfected with pAcGFP-C1-FPI proteins and incubated for 48 h.

FIGURE S2

FIGURE S2 IglE was secreted into culture medium *F. novicida* was cultured in BHIc medium containing 1% (a) or 5% (b) KCl. The bacterial cells were collected by centrifugation, and then the supernatants were filtered and desalted by ultrafiltration membrane. The membrane was treated with anti-IglE antiserum (a) or anti-PdpC antibody (b) (1:1000), followed by treatment with HRP-conjugated anti-rabbit IgG.
